# Whole genome sequence analysis of *Shigella* from Malawi identifies fluoroquinolone resistance

**DOI:** 10.1099/mgen.0.000532

**Published:** 2021-05-04

**Authors:** George E. Stenhouse, Khuzwayo C. Jere, Chikondi Peno, Rebecca J. Bengtsson, End Chinyama, Jonathan Mandolo, Amy Cain, Miren Iturriza-Gómara, Naor Bar-Zeev, Nigel A. Cunliffe, Jennifer Cornick, Kate S. Baker

**Affiliations:** ^1^​ University of Liverpool, Liverpool, UK; ^2^​ Malawi-Liverpool-Wellcome Trust Clinical Research Programme, College of Medicine, University of Malawi, Blantyre, Malawi; ^3^​ NIHR Health Protection Research Unit in Gastrointestinal Infections, University of Liverpool, Liverpool, UK; ^4^​ ARC Centre of Excellence in Synthetic Biology, Department of Molecular Sciences, Macquarie University, North Ryde, Australia; ^5^​ International Vaccine Access Center Department of International Health, Johns Hopkins Bloomberg School of Public Health, Baltimore, USA

**Keywords:** AMR, FQR, WGS, *Shigella*

## Abstract

Increasing antimicrobial resistance and limited alternative treatments have led to fluoroquinolone-resistant *Shigella* strain inclusion on the WHO global priority pathogens list. In this study we characterized multiple *Shigella* isolates from Malawi with whole genome sequence analysis, identifying the acquirable fluoroquinolone resistance determinant *qnrS1*.

## Data Summary

The authors confirm all supporting data, code and protocols have been provided within the article or through supplementary data files. The raw sequencing reads from the eight *Shigella* and *Escherichia coli* isolates which were subjected to whole genome sequencing have been deposited in the National Center for Biotechnology Information (NCBI) Short Read Archive under accession numbers: ERR2525592, ERR2525594, ERR2525595, ERR2525596, ERR2525597, ERR2525598, ERR2525599 and ERR252600. The accession numbers for all the reference *Shigella* and *E. coli* isolates used in the study are provided in the supplementary table.

**Impact Statement**

Antimicrobial resistance is a growing threat to our ability to treat bacterial infections. Multidrug resistance is widespread among *Shigella *, a leading cause of diarrhoeal death globally. The greatest disease burden from shigellosis is on those living in low- and middle-income nations in sub-Saharan Arica and Asia. Emerging resistance to fluoroquinolone, the recommended first-line treatment, has been identified in Asia but is probably being missed in understudied regions. This study characterizes eight shigellae from Malawi, an area where *Shigella* has been understudied, looking, in particular, at antimicrobial resistance. All the isolates were found to be multidrug resistant, with one isolate probably resistant to fluoroquinolones. This fluoroquinolone resistance element was found to be carried on a plasmid conferring resistance to several other antimicrobials, indicating a probable threat of dissemination of multidrug resistance in the region. This study highlights the importance of studying all high disease burden regions, to ensure ongoing effective treatment of diarrhoeal diseases.

## Introduction

*Shigella* is the second leading cause of diarrhoeal death globally with the greatest disease burdens seen in low- and middle-income countries [1]. Approximately one-third of these deaths are in children under the age of 5 years, with infection potentially causing chronic health effects [1, 2]. Antimicrobial resistance (AMR) increasingly limits treatment options, threatening to reverse hard won reductions in diarrhoeal mortality in high-burden areas [3]. Meanwhile, vaccines are still in development. There is therefore a need for effective public health solutions.

The increasing prevalence of fluoroquinolone-resistant (FQR) strains and limited, widely effective alternatives to this first-line treatment, has led to these strains being included on the WHO global priority pathogens list [4]. Resistance can be acquired *de novo* through a double mutation in the *gyrA* gene (amino acids 83 and 87) of the quinolone resistance determining region (QRDR), with a third mutation in the *parC* gene (AA80) ameliorating the fitness cost [5]. Resistance can also be acquired through horizontal transmission of FQR genes [5].

*Shigella* is reported as a leading cause of diarrhoea among hospitalized children in Malawi, but there is little information on the circulating strains [6]. Whole genome sequence analysis (WGSA) has been successfully applied to investigate *Shigella* epidemiology and AMR determinants, and can greatly aid in disease control in high-burden areas, such as Malawi [7]. Here, we have applied WGSA to characterize *Shigella* strains and AMR determinants in Malawi. Providing important baseline information for public health interventions including antibiotic treatment and deployment of *Shigella* vaccines, the development of which is a WHO priority [8].

### The study

All biochemically confirmed shigellae collected during a rotavirus vaccine evaluation programme between 2012 and 2015, isolated from faecal samples collected from children under the age of 5 years and hospitalized with acute gastroenteritis at the Queen Elizabeth Central Hospital, Blantyre, Malawi, were subjected to WGS (Supplementary methods) [9]. Species were confirmed with a maximum-likelihood phylogeny of the *Shigella */* Escherichia coli* clade, generated from a core single nucleotide polymorphism (SNP) alignment (40 075 SNPs) using quality trimmed reads mapped to a complete reference genome (Supplementary table). Only those genomically confirmed as *Shigella* or *E. coli* (8/10) were included in the study. All *Shigella* isolates were serotyped *in silico* with ShigaTyper (v1.0.6, https://github.com/CFSAN-Biostatistics/shigatyper).

**Fig. 1.**
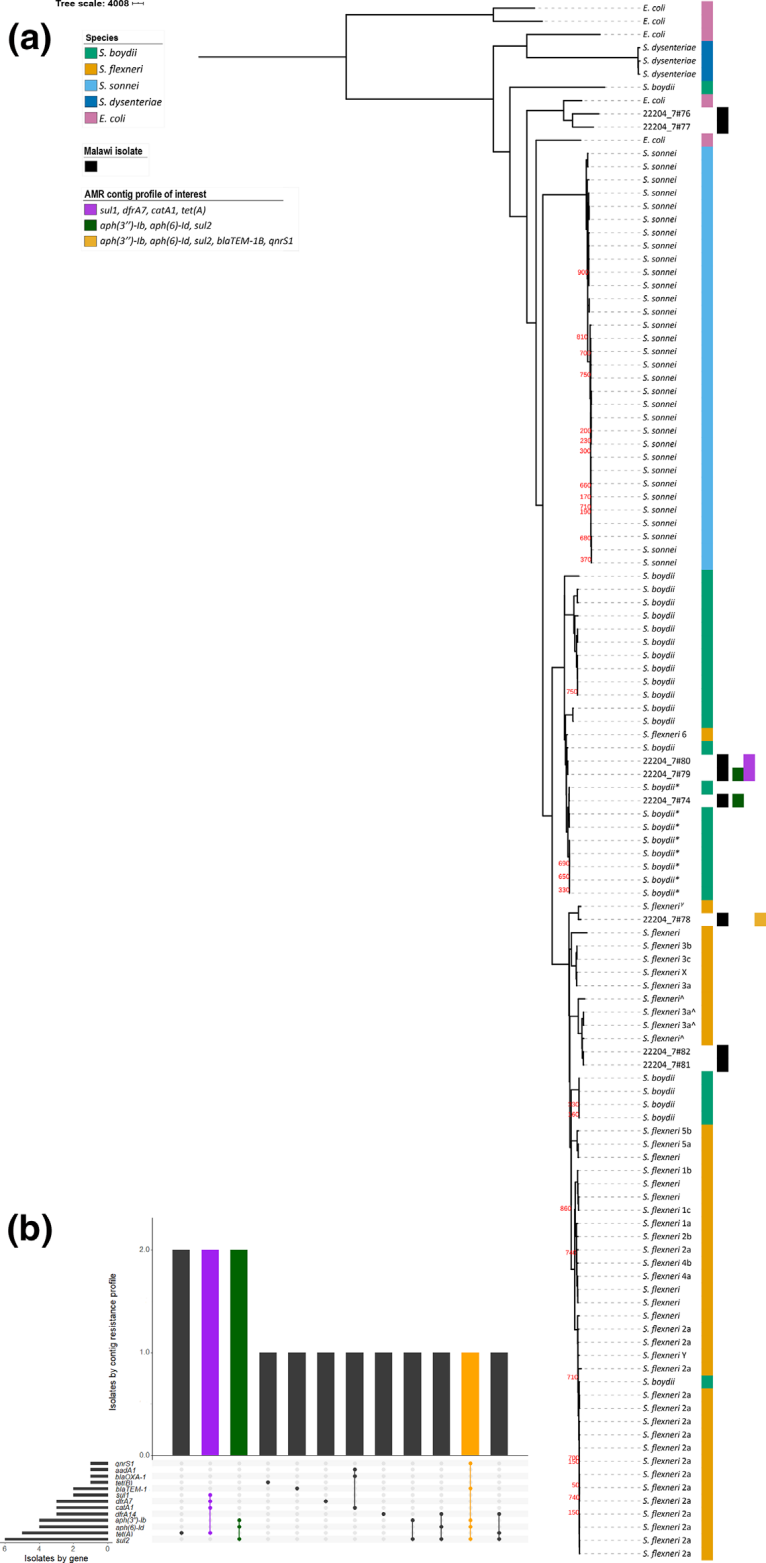
Maximum-likelihood phylogeny of eight isolates from Malawi, contextualized among *Escherichia coli* and *Shigella* and highlighting some AMR profiles of interest. AMR profiles of interest are those of contiguous sequences which are multi-drug-resistant and shared across multiple isolates, and in one isolate carries additional AMR genes including an FQR gene (*qnrS1*). They are indicated by columns to the right of the tree (**a**) and by coloured bars in the AMR profile chart (**b**). (**a**) Columns to the right of the phylogeny indicate, from left to right, species, study isolate and AMR profiles of interest. GTR+G substitution model, 1000 bootstrap validation and mid-point rooted. * S. boydii clade 3, ^* S. flexneri* phylogroup 2, ^γ^ *S. flexneri* phylogroup 7. All bootstrap values for internal nodes with support <900 are displayed. Bar, SNPs per site. (**b**) Intersection of individual AMR genes by isolate and AMR gene profile by contig.

From our eight isolates, four *Shigella* serotypes were identified: two *S. flexneri* 3a (Sf3a, Phylogroup 2), one *S. flexneri* 4av (Sf4av, Phylogroup 7), two *S. flexneri* 6 (Sf6) and one *S. boydii* 2 (Sb, Clade 3). Two isolates were of *E. coli* (Fig. 1a). The lack of *Shigella sonnei* isolates in our collection was expected as *S. sonnei *, though highly prevalent globally, is typically associated with high-income nations and industrialization [10]. Prevalence of *S. boydii* in Africa is thought to be low, isolated in 5.7 % of cases from a multi-national study [11]. However, detection with our small sample size would still be likely by chance (binomial test *P*=0.375) and may not be due to higher prevalence in Malawi. The strain diversity indicates that several, distinct strains circulate in Malawi; further characterization is needed to aid effective vaccine development for the region.

Identification of *E. coli* among the samples is probably due to the close relatedness between the two species. *Shigella* is a specialized pathovar of *E. coli *, sharing a disease phenotype with enteroinvasive *E. coli* (EIEC), which is highly adapted to humans [12]. To determine whether the *E. coli* isolates were EIEC we looked for the presence of the mxi-spa locus, found on the large virulence plasmid (pINV). This locus encodes a Type 3 Secretion System and secreted effector proteins which produce the distinctive invasive disease phenotype [12, 13]. Contiguous sequences (contigs) from quality-assessed isolate draft genomes, assembled using Unicycler (v0.4.7) [14], were compared against a reference EIEC pINV to identify locus presence (Supplementary table). Using this approach, one *E. coli* isolate was identified as EIEC (99 % identity and 100 % coverage with the mxi-spa locus).

As resistance is a growing hinderance to effective treatment, we determined genotypic AMR profiles for each isolate using starAMR (v0.5.1; https://github.com/phac-nml/staramr; Supplementary methods). All *Shigella* isolates were predicted to be multi-drug-resistant (MDR), carrying genes conferring resistance to three or more drug classes, demonstrating MDR *Shigella* circulate in Malawi (Table 1). While overall resistance was high, we observed limited diversity in AMR genes and predicted resistance profiles; 13 genes encoded resistance to seven antimicrobial classes (Fig. 1b, Table 1). This suggests that there are treatments which remain effective in Malawi, such as azithromycin, though this would need to be confirmed in a larger study and may change with mass drug administration programmes.

**Table 1.** AMR genotypic and predicted phenotypic profiles of Malawian *Shigella* isolates by contiguous sequence

Isolate ID	Contig length (bp)	Resistance genes	Predicted resistance drug class
22204_7#74 (* S. boydii* 2)	9131*	*tet(A*)	Tetracycline
	2798	*aph(3′′)-Ib, aph(6)-Id, sul2*	Aminoglycoside, sulfonamide
	2353	*dfrA7*	Trimethoprim
	1831	*blaTEM-1B*	Aminopenicillin
22204_7#78 (* S. flexneri* 4av)	48 082	*tet(A*)	Tetracycline
	17 334	*aph(3′′)-Ib, aph(6)-Id, sul2, blaTEM-1B, qnrS1*	Aminoglycoside, sulfonamide, aminopenicillin, fluoroquinolone
	1588	*dfrA14*	Trimethoprim
22204_7#79 (* S. flexneri* 6)	34 138	*tet(A), sul1, dfrA7, catA1*	Tetracycline, sulfonamide, trimethoprim, chloramphenicol
	6200	*aph(3′′)-Ib, aph(6)-Id, sul2*	Aminoglycoside, sulfonamide
22204_7#80 (* S. flexneri* 6)	25 733	*tet(A), sul1, dfrA7, catA1*	Tetracycline, sulfonamide, trimethoprim, chloramphenicol
	6200	*aph(3′′)-Ib, sul2*	Aminoglycoside, sulfonamide
22204_7#81 (* S. flexneri* 3a)	11 392†	*dfrA14, sul2, tet(A*)	Trimethoprim, sulfonamide, tetracycline
22204_7#82 (* S. flexneri* 3a)	45 377	*tet(B*)	Tetracycline
	8773	*aadA1, blaOXA-1, catA1*	Aminoglycoside, aminopenicillin, chloramphenicol
	6790‡	*aph(6)-Id, dfrA14, sul2*	Aminoglycoside, trimethoprim, sulfonamide

*StarAMR identified IncFIB(K) plasmid.

†StarAMR identified MDR IncQ1 plasmid.

‡Possible multi-copy plasmid.

**Fig. 2.**
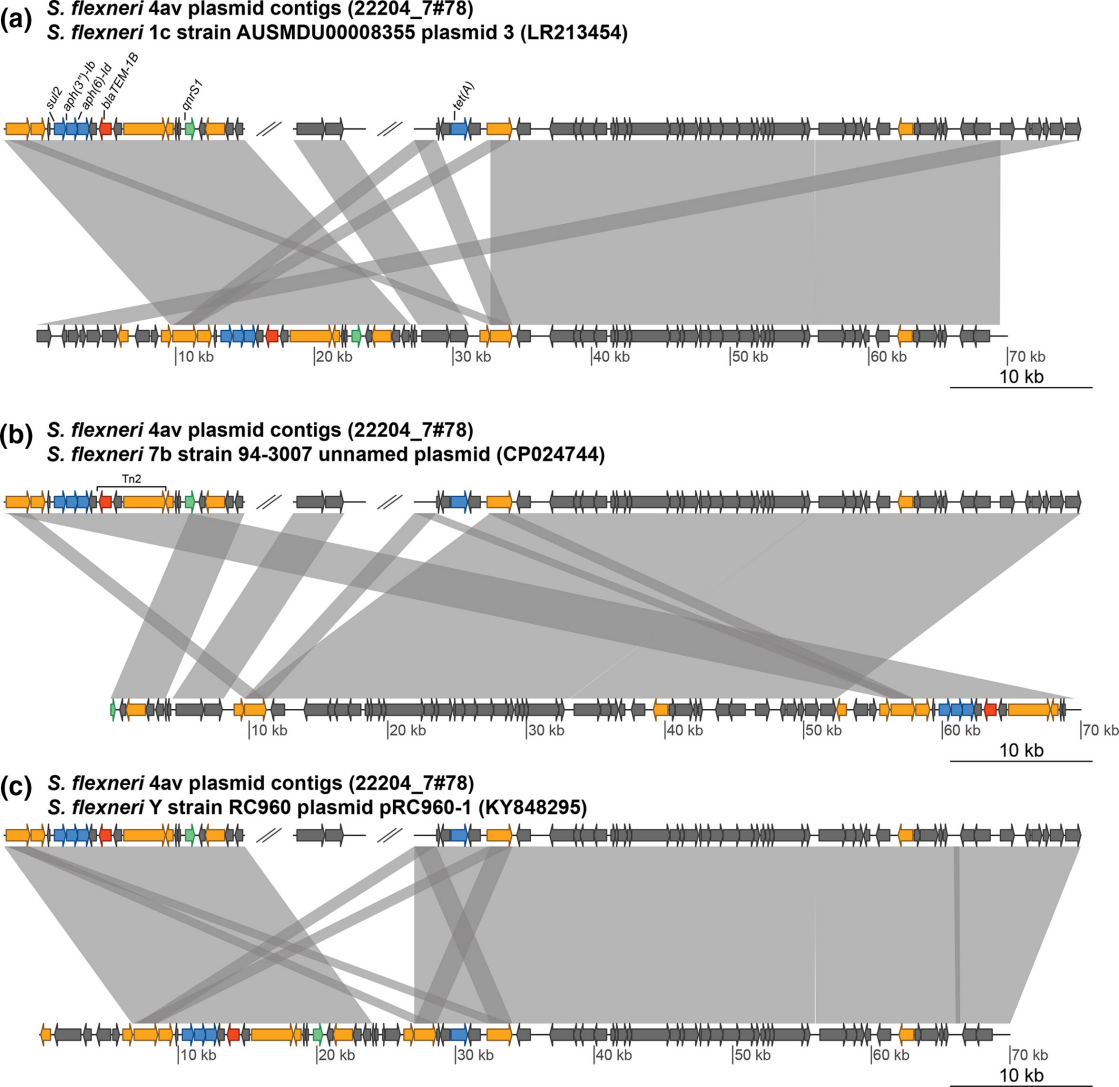
Pairwise comparisons of *S. flexneri* 4av study isolate plasmid contigs against previously identified MDR plasmids. Pairwise comparisons of the *S. flexneri* 4av isolate plasmid contigs (upper sequence) against the previously identified plasmids (lower sequences). Transposase genes are shown in orange and AMR genes in blue, except the FQR gene *qnrS1* which is in green and β-lactamase gene *blaTEM-1B* which is in red and is part of a Tn2 transposon. (**a**) Comparison against the *S. flexneri* 1c, strain AUSMDU00008355 plasmid 3, which lacks the *tet(A*) gene. (**b**) Comparison against the *S. flexneri* 7b, strain 94-3007 unnamed plasmid, which also lacks a *tet*(*A*) gene. (**c**) Comparison against the *S. flexneri* Y, strain RC690 plasmid pRC960-1.

Mobile genetic elements (MGEs) are important drivers of AMR dissemination, so we explored the genetic context of our AMR genes to look for evidence of them being within MGEs [15]. Resistance contigs from two isolates were identified as likely plasmid contigs, also using starAMR (Supplementary methods): one Sf3a isolate (22204_7#81) carried an MDR IncQ1 plasmid, and the Sf4av isolate carried a *tet(A*) encoding IncFIB(K) plasmid (Table 1). This contig was probably part of a MDR plasmid in combination with two other contigs, discussed below (Fig. 2). Another MDR contig had a read depth 5.29-fold higher than the chromosomal contigs, providing possible evidence of being part a multi-copy plasmid (Table 1). The same AMR gene profile (*aph(3′′)-Ib, aph(6)-Id* and *sul2*) was identified in contigs from multiple isolates (22204_7#74, 22204_7#78 and 22204_7#79) across distinct phylogroups (Sb2 clade 3, Sf4av Phylogroup 7 and Sf6 respectively), possibly indicating an MGE (Fig. 1, Table 1). A comparison of these contigs showed that they had similarity (≥99 % sequence identity) to only the AMR gene-encoding region (2620–3184bp) (Supplementary methods). In one case (Sb2), however, this was the entire contig. The limited similarity across the whole contigs, and the presence of insertion sequences and transposase genes surrounding the AMR gene-encoding region in the largest of these contigs (Sf4av), suggests that these genes are more likely to have been spread via transposition than carriage on plasmids (Fig. 2b). One AMR gene (*blaTEM-1B*) was identified as being carried within a Tn2 transposon (Fig. 2b). Together, our data support a role for MGEs in the spread of MDR *Shigella* in Malawi.

To predict FQR, we looked for the presence of either point mutations in the QRDR, or FQR-associated genes. Point mutations were identified by comparing the *gyrA* and *parC* amino acid sequences across all isolates, with amino acid identity at resistance-associated sites confirmed as expected based on the literature. One isolate (Sf4av) was predicted to be FQR, due to the presence of *qnrS1* (Table 1). A mutation at *parC* R91Q was also identified, but the same variation was present in all isolates, *E. coli* and *Shigella *, and probably represents natural variation rather than a resistance adaptation. Unfortunately, phenotype data were unavailable to confirm these findings.

Detection of *qnrS1* indicates acquirable FQR is present among *Shigella* in Malawi. As this gene is typically carried on a plasmid, a blast comparison of the *qnrS1-*containing contig (17 334 bases) against the NCBI nt database was performed [5]. Three top equivocal hits against *S. flexneri* plasmids (KY848295.1, CP024474.1, LR213454.1) were found (e-score=0.0, query sequence coverage and identity ≥99.9 %), suggesting that this gene might be carried on a related plasmid. To investigate this possibility, the contigs of the draft genome were compared (by blast) to identify other contigs which probably belong to this plasmid, and three contigs were identified in total (e-value=0.0) (Supplementary methods). Together, the contigs showed high sequence similarity and sequence coverage against all three plasmids, which were themselves highly similar to each other (Fig. 2). It is likely that our isolate was carrying a plasmid very similar to these other previously identified plasmids. This is supported by the isolate sequence read mapping coverage (mean depth=53 reads) to the *S. flexneri* 1c strain AUSMDU00008355 plasmid 3 (LR213454) (Supplementary methods). The probable presence of an *qnrS1*-carrying plasmid means a high risk of widespread FQR in Malawi and neighbouring regions; further study into the prevalence and nature of FQR in the region is needed.

All four of these highly similar plasmids are MDR, encoding *sul2, aph(3′′)-Ib, aph(6)-Id, blaTEM-1B* and *qnrS1* genes. Our isolate and one other also carry an additional resistance gene *tet(A*) (Fig. 2). Each was identified in a different *S. flexneri* serotype (1c, Y, 7b and 4av), suggesting the dissemination of this MDR plasmid across multiple lineages. They were also identified across multiple continents [specifically in Australia, China, America (unpublished) and Malawi] [16–18]. This suggests the plasmid has been successfully horizontally transmitted between strains and spread intercontinentally. It also provides evidence for an interaction between MDR *Shigella* globally and MDR *Shigella* in Malawi, though further research is needed to characterize this.

## Conclusions

Together, the high proportion of MDR *Shigella,* the acquirable FQR and the MDR plasmids detected in this study show that, without intervention, controlling shigellosis in Malawi will be increasingly difficult, which will probably have global consequences. We highlight the importance of further research into the epidemiology of *Shigella* in the region to ensure effective disease control.
